# Cross-Flow Catalysis Behavior of a PVDF/SiO_2_@Ag Nanoparticles Composite Membrane

**DOI:** 10.3390/polym10010059

**Published:** 2018-01-10

**Authors:** Wenqiang Wang, Xi Chen, Chu Zhao, Bowu Zhao, Hualin Dong, Shengkui Ma, Liying Li, Li Chen, Bin Zhang

**Affiliations:** 1 State Key Laboratory of Separation Membranes and Membrane Processes, Tianjin Polytechnic University, Tianjin 300387, China; wenqiang9109@163.com (W.W.); 18151308297@163.com (C.Z.); zbwnku@163.com (B.Z.); donghualin@tjpu.edu.cn (H.D.); mashengkui@tjpu.edu.cn (S.M.) chenli_tjpu@163.com (L.C.); 2 School of Environmental and Chemical Engineering, Tianjin Polytechnic University, Tianjin 300387, China; 3 School of Materials Science and Engineering, Tianjin Polytechnic University, Tianjin 300387, China; 4 Tianjin BeiAo Membrane Co., Ltd., Tianjin 300180, China; Tjzhangb@126.com

**Keywords:** Ag nanoparticles, catalysis, composite membrane, separation, SiO_2_ microspheres

## Abstract

A blend of Polyvinylidene Fluoride (PVDF) and SiO_2_ microspheres in *N*,*N*-Dimethylformamide (DMF) underwent phase inversion to form a PVDF/SiO_2_ membrane with SiO_2_ microspheres in the membrane’s pores. Subsequently, the SiO_2_ microspheres have been used as platforms for in site Ag nanoparticles (NPs) synthesis, forming a composite membrane. Benefitting from the full exposure of Ag NPs to the reactants, the composite membrane shows high catalytic reactivity when catalyzing the reduction of *p*-nitrophenol under a cross-flow. The catalytic reaction follows the first-order kinetics, and the reaction rate increases with an increase in the amount of Ag NPs in the membrane, the reaction temperature, and the operating pressure. What is more, highly purified products can be produced and separated from the reactants in a timely manner by using the composite membrane.

## 1. Introduction

Noble metal nanoparticles (NPs) are very attractive catalysts due to their highly active surface atoms [1,2,3]. However, they can easily agglomerate during the catalytic reaction process due to their high surface-to-volume ratio and surface energy [4]. In order to overcome this drawback in application, noble metal nanocatalysts are generally stabilized by stabilizers, such as polymers [2], complex ligands [5], and surfactants [4]. However, the catalytic reactivity of a metal nanocatalyst is low due to the restricted contact between the reactants and metal NPs resulting from the catalysts’ surfaces covered by these stabilizers [6,7,8]. Fortunately, many studies have indicated that metal NPs loaded onto the surfaces of SiO_2_ microspheres can fully expose their reactive points to reactants, and therefore have high reactivity [9,10,11,12]. However, the use and reuse of metal NPs is still difficult and inconvenient due to the aggregation of SiO_2_ microspheres [9,10].

Membranes have been strongly suggested as supports for metal catalysts [13,14,15,16]. Membranes have large surface areas for loading metal NPs and many opened pores for reactants’ passage. Furthermore, membranes with metal catalysts can be used continuously and repeatedly, without the need to separate the metal catalysts from the reaction system [13,14,15,16,17]. In addition, membranes as platforms can enhance the circular stability and longevity of the metal catalysts [13,17]. However, a tedious process is needed to introduce metal NPs into a membrane because the commonly used membrane material has no reactive groups for binding metal NPs [15,18]. Recently, we facilely introduced noble metal NPs into a membrane by introducing polymeric spheres into the same one beforehand for loading metal NPs [13,17]. However, the functional polymers for binding metal NPs have been easily swollen, leading to a negative effect on catalysis [2,15,16,18]. Therefore, it is necessary to find a better strategy to load the noble metal NPs onto a membrane.

In addition, membranes have two external surfaces and many inner pores, which play different roles in a filtration process [19]. By the rejection of the membrane surface and the selective permeability of the membrane pore, different substances are separated from a mixture [19,20]. However, for too long, catalytic membranes have been only used as catalysts and their separation performance has not been focused on a catalysis process [15,16,18]. Even so, it is clear that the timely separation of products from reactants is still very important and necessary when a membrane is used as a catalyst because it can significantly decrease the operation’s cost and avoid the undesired side reaction possible from the reactants and products [13,16,21]. Previous reports have provided many successful technologies to anchor metal NPs into a polymeric membrane, but the formed membrane cannot realize the desired timely separation of products due to the metal catalysts both on the membrane’s surface and in the membrane’s pores, which always leads to a mixture of reactants and products [15,16,18]. Obviously, a new membrane is needed to realize this special separation property [22]. Recently, we reported the successful separation of products from reactants by using a composite membrane with polymeric spheres and embedded metal NPs in the membrane’s pores [13,17]. Since these polymeric spheres have the intrinsic drawbacks of wrapping the metal catalysts [2], they should be replaced by a better candidate.

Here, we report a composite membrane with SiO_2_ microspheres coated by Ag NPs in the membrane pores and its catalytic property. The composite membrane was prepared from a first formation of a blend membrane with a PVDF substrate and SiO_2_-NH_2_ microspheres, and the subsequent growth of Ag NPs on the surfaces of the SiO_2_-NH_2_ microspheres. The catalysis and separation properties of the novel membrane were evaluated by the reduction of *p*-nitrophenol under a cross-flow model. The Ag NPs have high catalytic reactivity and a relatively low cost among the noble metal nanocatalysts, and the reduction of *p*-nitrophenol is a simple way for producing *p*-aminophenol, which is an important fine chemical intermediate [4,8,9]. Therefore, this reaction system will, no doubt, have significant value for the chemical industry.

## 2. Experimental Section

### 2.1. Materials and Reagents

Tetraethylorthosilicate (TEOS, 98%) and 3-(Aminopropyl)trimethoxysilane (APTMS, 97%) were purchased from Aladdin Chemistry Co., Ltd., Shanghai, China. Aqueous ammonia (NH_3_·H_2_O, 25%), anhydrous ethanol (99.7%), silver nitrate (AgNO_3_, 99.8%), and *p*-nitrophenol, sodium borohydride (NaBH_4_, 98%) were supplied by Kermel Reagent Co., Ltd., Tianjin, China. PVDF powders (*M*_w_ = 3.52 × 10^5^, *M*_w_/*M*_n_ = 2.3, Solvay Company, Brussels, Belgium, Solef 1010) were used as received. *N*,*N*-Dimethylformamide (DMF, 99.5%) and polyvinylpyrrolidone (PVP K30, *M*_w_ = 58,000) were purchased from Guang Fu Fine Chemical Research Institute (Tianjin, China). All of the reagents were of analytical grade and used without further purification.

### 2.2. Synthesis of SiO_2_-NH_2_ Microspheres

SiO_2_ microspheres were synthesized by the Stöber method [23] and then modified with -NH_2_ groups through APTMS. In brief, a solution of ammonia (25.0 mL) and anhydrous ethanol (200.0 mL) was added into a three-neck round-bottom flask (500.0 mL) by vigorous stirring at 25 °C. After 0.5 h, TEOS (10.0 mL) was added to the flask. After another 12.0 h, APTMS (0.5 mL) was introduced into the solution. The mixture was retained at 80 °C for 2.0 h and then centrifuged. The SiO_2_-NH_2_ microspheres were obtained after the solid product underwent three cycles of centrifugation, re-suspension in anhydrous ethanol, and a final drying at 60 °C.

### 2.3. Preparation of the Composite Membrane

Preparation of the PVDF/SiO_2_ blend membrane: the PVDF/SiO_2_ blend membrane was prepared by immersion precipitation phase inversion [19,20]. PVDF powders (6.80 g), the synthesized SiO_2_-NH_2_ microspheres (1.20 g), and PVP (0.47 g) were dispersed in DMF (41.42 mL) by vigorous stirring until a clear homogeneous solution was obtained at 60 °C. After being degassed under vacuum, the casting solution was cast onto a glass plate, which was subsequently immersed in de-ionized water (25 °C). When the nascent membrane was separated from the substrate, it was rinsed with de-ionized water to remove the residual solvent thoroughly and then stored in de-ionized water.

Preparation of the composite membrane: in a dark environment, the as-prepared PVDF/SiO_2_ membranes were immersed into different AgNO_3_ solutions (2.18 × 10^−3^, 2.18 × 10^−4^, and 2.18 × 10^−5^ mM) for 24.0 h to load Ag^+^ onto SiO_2_-NH_2_ microspheres of membranes. After the unbound Ag^+^ in a membrane was washed by de-ionized water, fresh NaBH_4_ solution was then used to reduce the Ag^+^ of the membrane into Ag NPs. The obtained PVDF/SiO_2_@Ag composite membranes were further rinsed with de-ionized water and then kept in de-ionized water until use. By changing the concentration of AgNO_3_ solution, three composite membranes were prepared, named as MB-Ag-1, MB-Ag-2, and MB-Ag-3, respectively.

### 2.4. Characterization

#### 2.4.1. Characterization of Composition, Structure, and Morphology

Fourier Transform Infrared Spectra (FTIR) of the SiO_2_ microspheres and SiO_2_-NH_2_ microspheres were recorded using a Bruker VECTOR-22 IR spectrometer (Bruker Daltonic Inc., Karlsruhe, Germany). The chemical composition of a membrane was characterized using attenuated total reflectance Fourier Transform Infrared Spectra (ATR-FTIR) with Zinc Selenide (ZnSe) as an internal reflection element at an incident angle of 45°. The spectra were collected at 16 scans at a resolution of 4.0 cm^−1^ and recorded in a wave number range of 4000–400 cm^−1^.

X-ray photoelectron spectroscopy (XPS) measurements of membrane surfaces were performed by using a Thermo Fisher K-alpha X-ray photoelectron spectrometer (Thermo Scientific, Waltham, MA, USA) with a monochromated Al Kα X-ray source (1486.6 eV photons) at a pass energy of 93.9 eV. The measurements were conducted at a take-off angle of 45° with respect to the sample surface. Survey XPS spectra were obtained by sweeping over 0–1350.0 eV electron binding energy with a resolution of 1.0 eV.

The membranes’ morphologies were observed by field emission electron microscopy (FESEM, Hitachi S-4800, Tokyo, Japan). Dried samples were freeze-fractured using liquid nitrogen and then sputter-coated with a thin gold layer to increase the contrast and quality of the images. An energy dispersive X-ray analysis (EDX) was conducted to examine the cross-sectional compositions of the composite membranes.

#### 2.4.2. Water Contact Angle Measurement

Water contact angle measurements were performed with the sessile drop method using a contact angle meter (JYSP-180, Jinshengxin, Testing Machine Co., Beijing, China) [24]. A syringe with a needle diameter of 0.525 mm was used to place a water droplet of 4.0 μL on the membrane surface. The contact angle was measured by generating tangent lines to both sides of the droplet static image with the Drop Shape Analysis software.

### 2.5. Measurements of a Membrane’s Porosity and Pore Size

The porosity (ε) of a membrane was determined at 25 °C by the wet-dry weighting method [25]. The porosity was calculated as the pore’s volume divided by the membrane’s volume with Equation (1) [13]:

(1) $$\varepsilon =\frac{W_{1}-W_{2}}{\rho_{w}\times A\times l}\times 100\%,$$

where *W*_1_ (g) is the weight of the wet membrane, *W*_2_ (g) is the weight of the dry membrane, *l* (mm) is the membrane’s thickness, *A* (cm^2^) is the effective membrane surface area, and $\rho_{w}$ (g·cm^−3^) is the water density. The average pore size (*d*) of the membrane was calculated according to the pure water flux by the Guerout-Elford-Ferry equation [20]:

(2) $$d=\sqrt{\frac{(2.9-1.75\varepsilon )\times 8\eta lJ}{\varepsilon \times A\times \Delta P}},$$

where ε is the membrane’s porosity, η (Pa·s) is the pure water viscosity, Δ*P* (MPa) is the operation pressure, and *J* (L·m^−2^·h^−1^) is the volume of pure water penetrating through the membrane during unit time.

### 2.6. Measurement of Metal Content

Metal contents were determined by measuring the concentrations of metal ions of a dilute nitric acid extract of the metal NPs of the composite membrane using inductively coupled plasma atomic emission spectrometry (ICP-AES, Varian 715-ES, Palo Alto, CA, USA). Before measurement, the composite membrane was immersed in nitric acid (10.0 mL, 5.0 M) for 0.5 h and then diluted to a final volume of 100.0 mL by de-ionized water.

### 2.7. Pure Water Flux through a Membrane

A membrane (17.9 cm^2^) was firstly pre-pressured for 1.0 h under an operating pressure to maintain a steady state. Then, the pure water flux was measured under a cross-flow pattern [24,25]. The water flux (*J*) was obtained by an average of three measurements according to Equation (3) [24]:

(3) $$J=\frac{Q}{A\times \Delta t},$$

where *Q* (L) is the permeate volume and Δ*t* (h) is the filtration time.

### 2.8. Catalytic Reduction of p-Nitrophenol by a Composite Membrane

**Figure 1:**
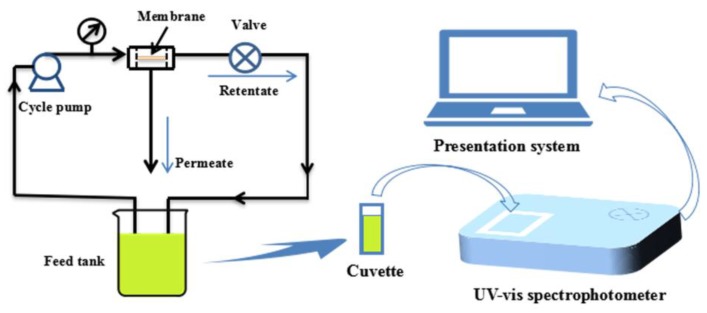
The diagram of experimental setup for catalysis.

The reactivity of the prepared PVDF/SiO_2_@Ag composite membrane was investigated by the reduction of *p*-nitrophenol to *p*-aminophenol. The reaction was carried at pH 10.0. A feed solution was prepared by mixing a *p*-nitrophenol solution (0.144 mM, 490.0 mL) with a freshly prepared NaBH_4_ aqueous solution (79.22 mM, 10.0 mL). The catalytic reaction was carried out under a cross-flow experiment apparatus as shown in Figure 1. Under a cross-flow, the feed solution circularly flowed through a composite membrane (*A* = 17.9 cm^2^ and *l* = 0.16 mm) at 0.1 MPa and 25 °C. The concentration change of *p*-nitrophenol in the feed solution was studied by monitoring the absorbance maximum (λ_max_ = 400 nm) of *p*-nitrophenol with a UV-Vis spectrophotometer (TU-1810PC, Beijing Purkinje General Instrument Co., Ltd., Beijing, China) at an interval of 2.0 min.

### 2.9. Reusability Test

The catalytic recyclability was determined by repeating the measurement of *p*-nitrophenol reduction described in Section 2.8 with a composite membrane. After one measurement cycle, the membrane was washed thoroughly with water and the next catalytic run was then started. The aforementioned procedure was repeated eight times.

## 3. Results and Discussion

### 3.1. Characterizations of SiO_2_ and SiO_2_-NH_2_ Microspheres

**Figure 2:**
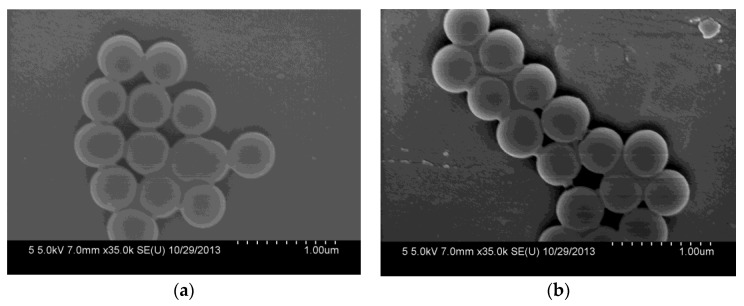

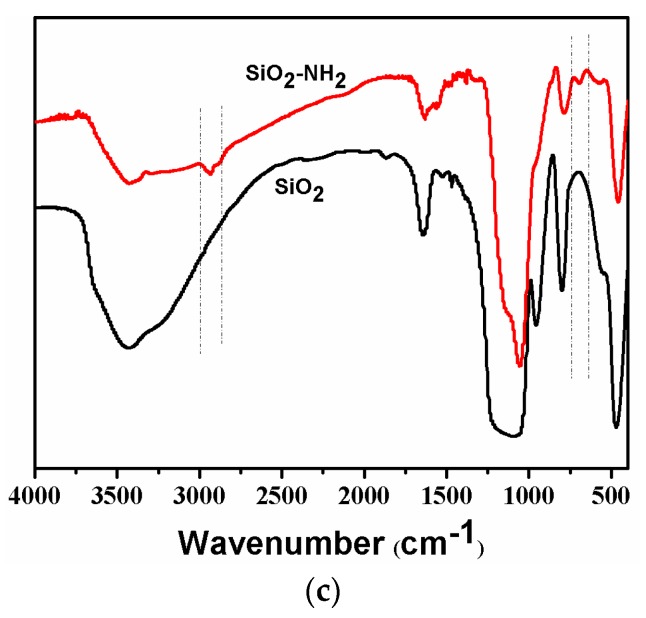
Field emission electron microscopy (FESEM) of synthesized SiO_2_ microspheres (**a**) and SiO_2_-NH_2_ microspheres (**b**) and their FTIR spectra (**c**).

The morphologies of the synthesized SiO_2_ and SiO_2_-NH_2_ are observed by FESEM. As shown in Figure 2a, the as-prepared SiO_2_ is a uniform microsphere. The average diameters of the SiO_2_ microspheres are about 400.0 ± 20 nm. Meanwhile, the SiO_2_-NH_2_ also maintains a spherical shape, and does not show any difference to SiO_2_ (Figure 2b). Figure 2c shows the FT-IR spectra of SiO_2_ and SiO_2_-NH_2_ microspheres. All of the microspheres show peaks at around 1095 and 802 cm^−1^, which are attributed to the Si–O–Si vibration [26]. The bands at 3500–3200 and 1630 cm^−1^ correspond to the Si–OH stretching [10,12,27]. Compared to the spectrum of the SiO_2_ microsphere, the new peaks in the ranges of 3000~2850 and 730–650 cm^−1^ appearing in the spectrum of the SiO_2_-NH_2_ microsphere correspond to the C–H stretching vibration and N–H bending vibrations (APTMS), respectively [3]. The results demonstrate the presence of -NH_2_ on the surfaces of SiO_2_ microspheres, which can be used in the loading of Ag NPs for catalysis [9,10].

### 3.2. Chemical Composition of a Membrane

**Figure 3:**
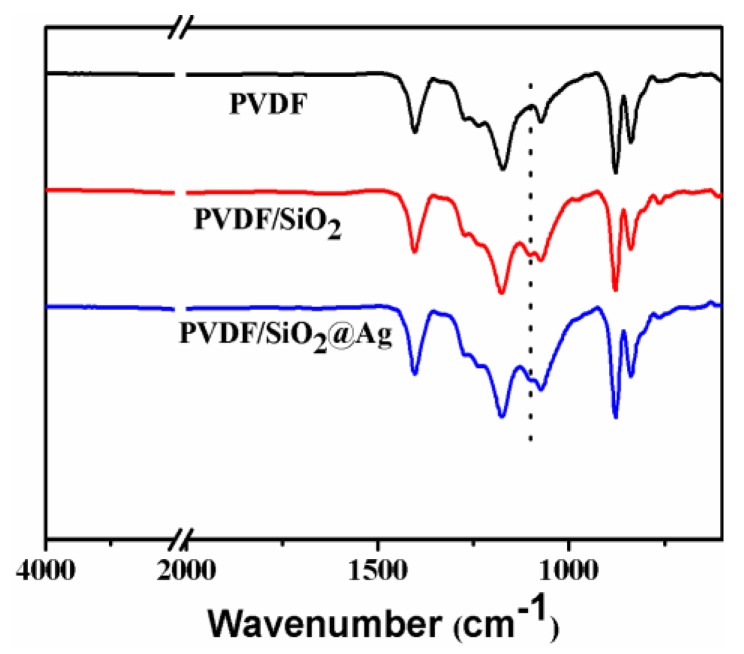
Transform Infrared Spectra (ATR-FTIR) spectra of the pure PVDF membrane, the PVDF/SiO_2_ membrane, and the PVDF/SiO_2_@Ag membrane: MB-Ag-1.

Figure 3 shows the ATR-FTIR spectra of the membranes. For all membranes, strong peaks at around 1280–1110 and 1461–1346 cm^−1^ are observed, which are assigned to the CF_2_ and CH_2_ of PVDF [20,24,25]. In comparison with the PVDF membrane, the PVDF/SiO_2_ and PVDF/SiO_2_@Ag membranes show distinct absorption bands of Si–O–Si at 1095 and 802 cm^−1^ (Figure 3) [9,10,12,26], confirming SiO_2_-NH_2_ microspheres in the blend membrane and composite membrane. Because of the very small content, the –NH_2_ cannot be examined by the FTIR, but can be found by XPS measurement.

**Figure 4:**
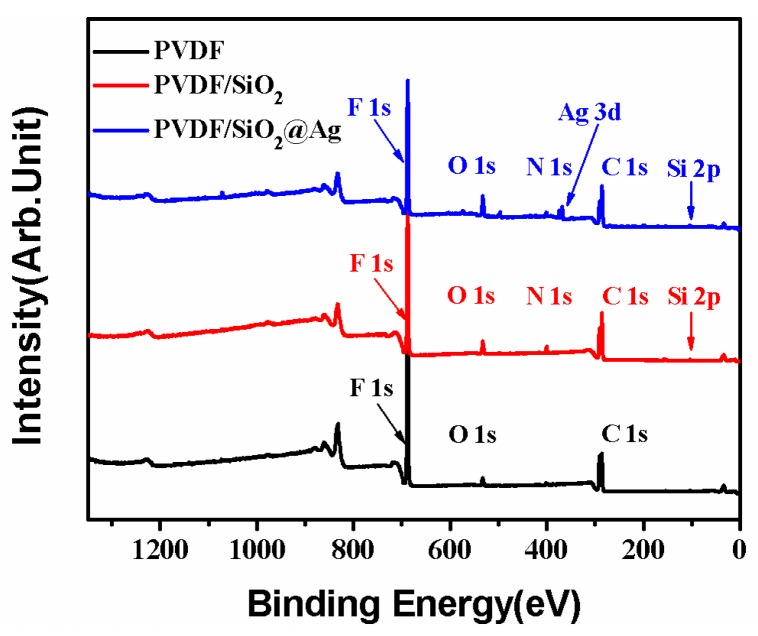
X-ray photoelectron spectroscopy (XPS) survey spectra of the pure PVDF membrane, the PVDF/SiO_2_ membrane, and the PVDF/SiO_2_@Ag membrane: MB-Ag-1.

As shown in Figure 4, the PVDF/SiO_2_ membrane shows all the binding energies that are ascribed to the PVDF membrane. Besides, the PVDF/SiO_2_ membrane also shows a new binding energy (BE) at 100.5 eV that is assigned to Si–O–Si [28], and a new one at about 397.8 eV that is assigned to the -NH_2_ groups of SiO_2_-NH_2_ [3]. Compared with the PVDF/SiO_2_ membrane, the peaks corresponding to Ag 3d at BEs of 368.0 and 374.0 eV are also observed in the XPS spectra of the PVDF/SiO_2_@Ag membrane, indicating that Ag^0^ species have been loaded onto the composite membrane [29]. Thus, the XPS spectra confirm the presence of SiO_2_-NH_2_ microspheres and Ag^0^ in the PVDF/SiO_2_@Ag membrane.

### 3.3. Morphology and Structure of the Membrane

**Figure 5:**
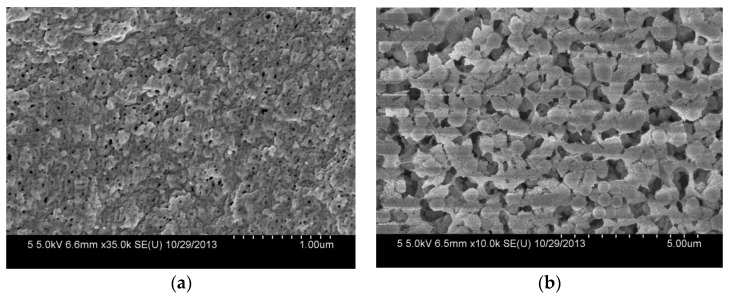
FESEM images of the top surface (**a**) and the bottom surface (**b**) of PVDF/SiO_2_@Ag membranes: MB-Ag-1.

The morphologies of the composite membranes are observed by FESEM. As shown in Figure 5a, the composite membrane has a porous top surface with a pore size of about 20.0 nm. The small surface pores are formed by the instantaneous phase separation of the casting solution on the glass plate. Compared with the top surface, the bottom surface of the composite membrane shows rough and larger pores (Figure 5b), which are caused by the slow diffusion between the solvent and nonsolvent in the membrane formation process due to the bottom surface being closed to the glass plate [20].

**Figure 6:**
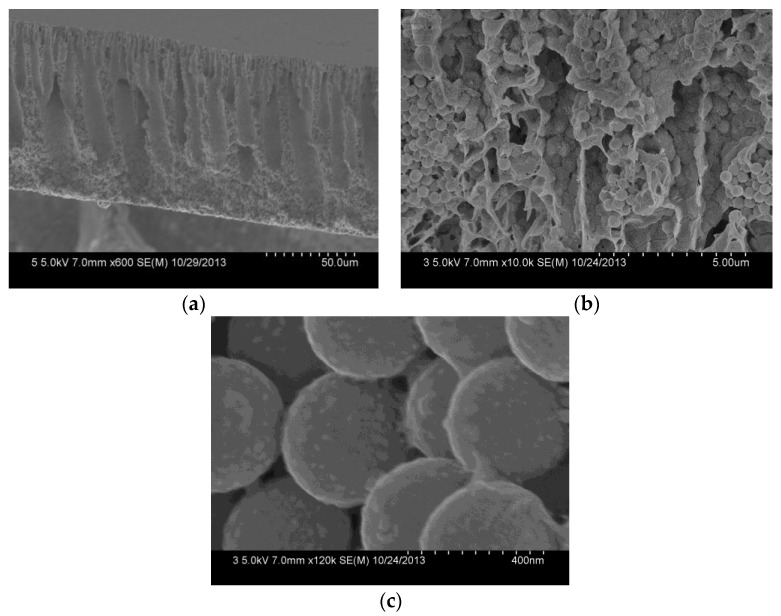
FESEM images of the cross-section (**a**), the further amplified cross-section (**b**) and the SiO_2_ microspheres (**c**) of the PVDF/SiO_2_@Ag membrane: MB-Ag-1.

The cross-section of the composite membrane shows a typical unsymmetrical structure, including a dense separation layer on the top surface of the membrane, a finger-like middle layer. And a sponge-like bottom layer (Figure 6a). The dense layer can resist protein or pollutants, and thereby reduce their pollution of the membrane inner in the filtration process. Due to the phase separation resulting from the poor interfacial compatibility between the SiO_2_-NH_2_ spheres and the PVDF in the membrane formation, the middle layer forms many pores with several thousand nanometers, which provide sufficient space for the SiO_2_-NH_2_ spheres used for binding metal catalysts [30]. The cross-section of the composite membrane (Figure 6b) shows that SiO_2_-NH_2_ microspheres are uniformly distributed in the membrane pores. A further amplified image (Figure 6c) shows that Ag NPs with a size of about 30.0 ± 5 nm are clearly stabilized on the surfaces of SiO_2_-NH_2_ microspheres. Because of the much larger sizes of the SiO_2_-NH_2_ microspheres than those of the surface pores, Ag NPs coated on the SiO_2_-NH_2_ microspheres can be long-term stained in the membrane pores, which is profitable for a catalysis application.

**Figure 7:**
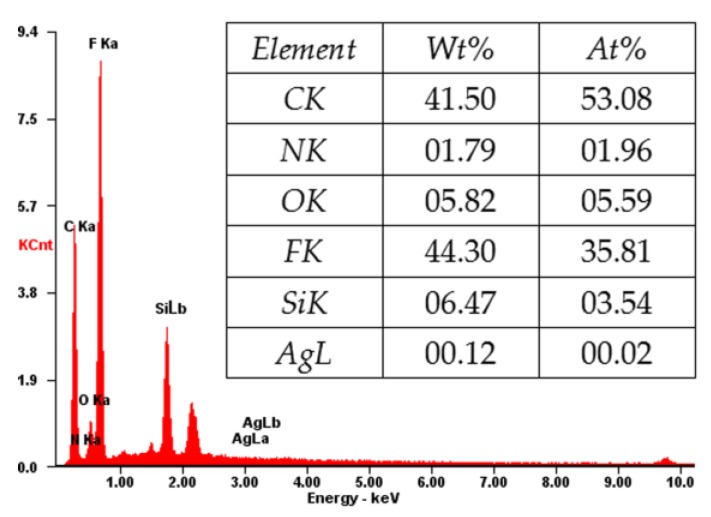
X-ray analysis (EDX) of the PVDF/SiO_2_@Ag membrane: MB-Ag-1.

Figure 7 shows the EDX spectrum of the cross-section of the composite membrane. As shown, the elements of C, N, O, F, Si, and Ag coexist, which is consistent with the XPS result (Figure 4). The content of the F and C elements is high, indicating that the main component of the composite membrane is PVDF [31], which will provide a good mechanical property for the composite membrane. The N and Si elements indicate the amino-functionalized SiO_2_ microspheres in the membrane, which will account for the loading of Ag NPs [9,10,32]. The successful loading of Ag is confirmed by the Ag element in the EDX spectrum (Figure 7). The loaded Ag content is measured by ICP-AES. As shown in Table 1, the Ag loading content of the membrane increases from 0 to 5.32, 16.79, and 59.96 μg/cm^2^ by increasing the concentration of AgNO_3_ (2.18 × 10^−5^, 2.18 × 10^−4^, and 2.18 × 10^−3^ mM, respectively) in the preparation process.

The porosity, average pore size, and water contact angle of various membranes are also shown in Table 1. As can be seen, the incorporation of Ag NPs hardly changes the porosity, pore size, and water contact angle of the PVDF/SiO_2_@Ag composite membrane.

### 3.4. Catalytic Property

#### 3.4.1. Catalytic Kinetics

**Figure 8:**
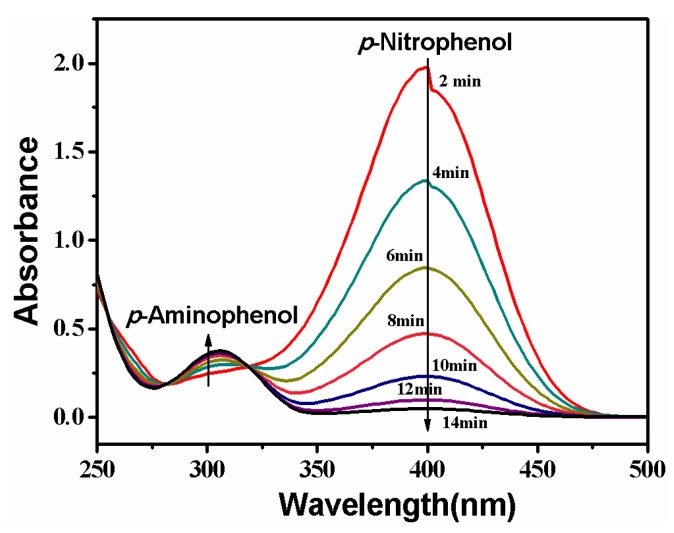
Change of UV-Vis absorption of the feed solution. Membrane: MB-Ag-1.

Reduction of *p*-nitrophenol is catalyzed by the PVDF/SiO_2_@Ag composite membrane. The process is monitored by continuously recording the UV-Vis absorption spectrum of the feed solution, and the result is shown in Figure 8. It can be seen that a strong absorption peak at 400 nm appears at first. This is because of the formation of *p*-nitrophenol ions in an alkaline condition [2,8,11]. The peak at 400 nm will remain unaltered in the absence of any catalyst [11,13,33]. However, when the feed solution circularly flows through the composite membrane, the peak at 400 nm gradually decreases with time. At the same time, the feed solution fades from the pale yellow color and ultimately becomes colorless. In this process, a new peak appears at 310 nm, which is ascribed to the absorption of the product of *p*-aminophenol [2,33]. The peak strength weakly increases with time, suggesting that *p*-nitrophenol is gradually converted to *p*-aminophenol. In the UV-Vis absorption spectrum, two isosbestic points are always seen at 280 and 314 nm, indicating that only *p*-aminophenol is formed during this reaction [2,8,11,13]. The reaction rapidly finishes at a short time of about 14.0 min due to large quantities of reactants accessing Ag NPs at unit time under the cross-flow model.

The composite membrane belongs to a heterogeneous catalyst [14,15,16,18,34]. It is well-known that the heterogeneous catalytic reaction model follows the Langmuir-Hinshelwood model, because the reaction is based on the adsorption of reactants on the catalyst surface [14,15,16,18,35]. The conversion of the reaction can be derived from the *C_t_*/*C*_0_, measured by the relative intensity of UV-Vis absorbance at 400 nm. Herein, *C_t_* (mM) is the concentration of *p*-nitrophenol at the reaction time *t* and *C*_0_ (mM) is the initial concentration. Since the dose of NaBH_4_ is greatly excessive (see Section 2.8), the reaction process can be described by the following first-order kinetic equation [2,9,35].

(4) $$-\ln(\frac{C_{t}}{C_{0}})=k_{obs}t=K_{SA}a_{s}\rho_{m}t$$

Herein, *k_obs_* is the apparent rate constant, reflecting the reaction rate, and *K_SA_*, *a_s_*, and *ρ_m_* are the surface area-based rate constant (L·min^−1^·m^−2^), the specific surface area of Ag NPs (m^2^·g^−1^), and the mass concentration of Ag NPs (g·L^−1^), respectively.

**Figure 9:**
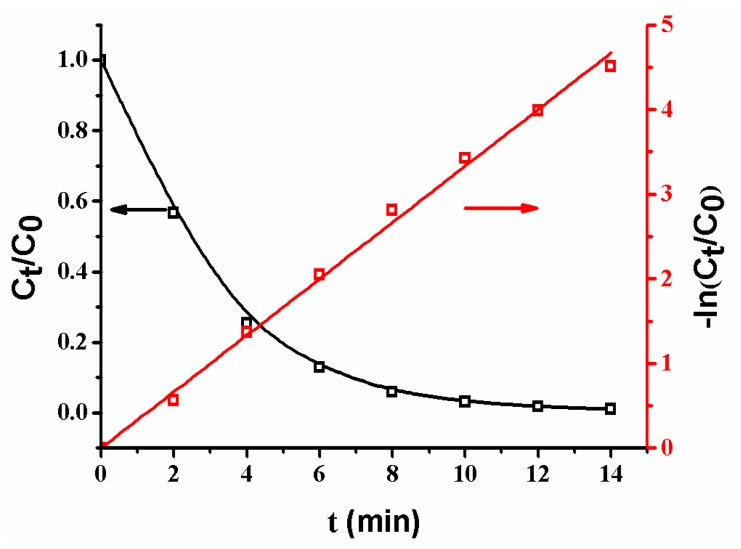
Changes of *C_t_*/*C*_0_ and ln(*C_t_*/*C*_0_) with time. Membrane: MB-Ag-1.

The plots of *C_t_*/*C*_0_ and −ln(*C_t_*/*C*_0_) versus time are shown in Figure 9. It is obvious that *C_t_*/*C*_0_ decreases with an increase of reaction time, revealing that the concentration of *p*-nitrophenol gradually decreases [2,8,10,13,17]. Additionally, the plot of −ln(*C_t_*/*C*_0_) versus reaction time yields a good linear relation in the reaction, indicating that the reaction follows the first-order kinetics [2,12,13,17,18]. The reaction rate constant (*K_SA_* = 33.2 L·min^−1^·m^−2^) is faster than that (*K_SA_* = 27.9 L·min^−1^·m^−2^) of the reaction catalyzed by a composite membrane with Ag NPs coated on the surfaces of poly (methacrylic acid) (PMAA) microspheres in membrane pores [13]. The different reaction rates are ascribed to the different composite membrane structures. When Ag NPs are coated on the surfaces of PMAA spheres, the swelling of PMAA spheres by water could cause a wrap of Ag NPs by polymer chains, leading to a low reaction rate; in contrast, as Ag NPs are coated on the surfaces of SiO_2_ spheres, the groups on the surfaces of SiO_2_ spheres are so short that they cannot wrap the Ag NPs [9,10,11,12]. Thus, the as-prepared composite membrane exhibits a high reaction rate.

#### 3.4.2. Effect of the Initial Concentration of *p*-Nitrophenol

**Figure 10:**
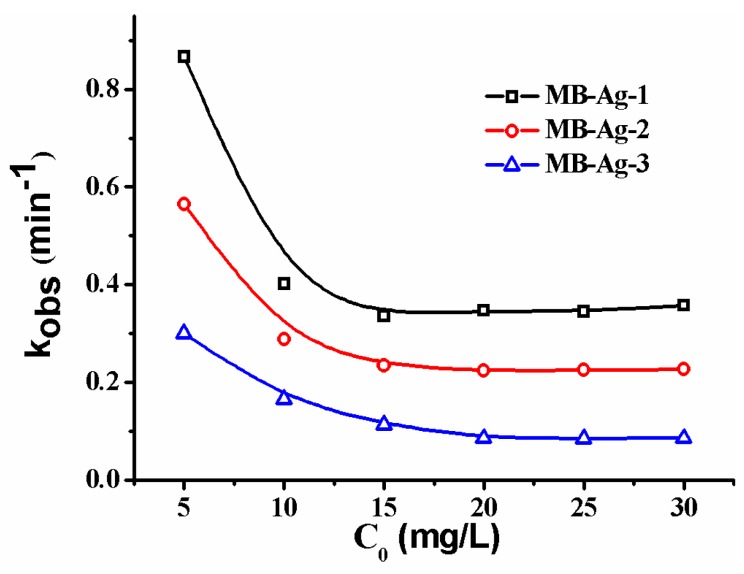
Effect of the initial concentration of *p*-nitrophenol on catalytic rate.

Figure 10 shows the *k_obs_* of the composite membrane under different *p*-nitrophenol concentrations. As can be seen, the *k_obs_* decreases with an increase in the initial *p*-nitrophenol concentration in a concentration range of 0.036–0.108 mM. In such a concentration range, the increase of *p*-nitrophenol causes a slower decrease of *C_t_*/*C*_0_ with time, leading to the decline of *k_obs_*. However, as the initial concentration of *p*-nitrophenol is higher than 0.1 mM (0.144–0.216 mM), the increase of *p*-nitrophenol only weakly affects the change of *C_t_*/*C*_0_ with time, and thus the *k_obs_* tends to a low constant value. As is well-known, the reaction occurs on the Ag NPs’ surfaces by transferring electrons from NaBH_4_ to *p*-nitrophenol [4,14,35]. Therefore, the synergistic effects of the rate of electron transfer at the metal surface and the diffusion of *p*-nitrophenol to the metal surface, together with the rapid diffusion of *p*-aminophenol away from the surface, should be responsible for the catalytic rate.

#### 3.4.3. Effect of Operating Pressure

**Figure 11:**
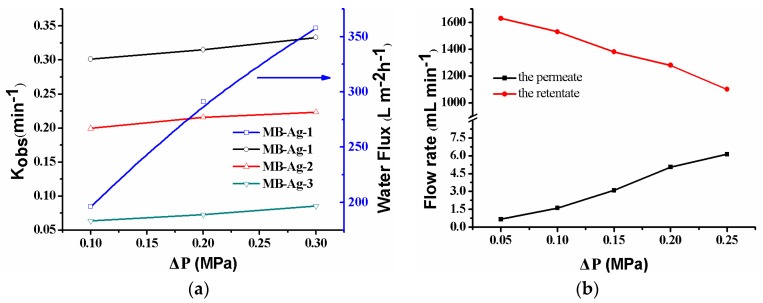
Effect of pressure on the catalytic rate, the water flux (**a**), and the flow rate (**b**) for the *p*-nitrophenol reduction with the MB-Ag-1 membrane.

The catalytic reaction is directly related to the flow rate of reactants controlled by the operating pressure. Figure 11a shows the effect of the operating pressure on the catalytic reaction. As shown, when the operating pressure varies from 0.10–0.30 MPa, the *k_obs_* increases. Under the present condition, the water flux linearly increases with an increase in the operating pressure, indicating that the composite membrane has good compression performance. The *k_obs_* increase is due to the flow rate change resulting from the increase in the operating pressure. As shown in Figure 11a, the water flux increases with an increase in the operating pressure, leading to the shorter residence time of the feed solution inside of the membrane. Thus, the feed solution can quickly contact the Ag NPs in the membrane. At the same time, reaction product can also leave the membrane more rapidly. The two factors contribute to the increase of the catalytic reaction rate [13,17]. On the other hand, an increase in operating pressure will cause a decrease in the flow rate of the feed solution (Figure 11b) and an increase in the residue time of the feed solution on the membrane’s surface. However, the flow rate of the feed solution on the membrane’s surface is still very large (Figure 11b) and does not affect the rapid contact of reactants with Ag NPs in the mouths of the membrane pores. Thus, an increase in the operating pressure is also beneficial to the reaction in this case. Therefore, the catalytic rate shows an increase as a result of increasing the operating pressure.

#### 3.4.4. Catalytic Activity as a Function of Ag Coating Content

**Figure 12:**
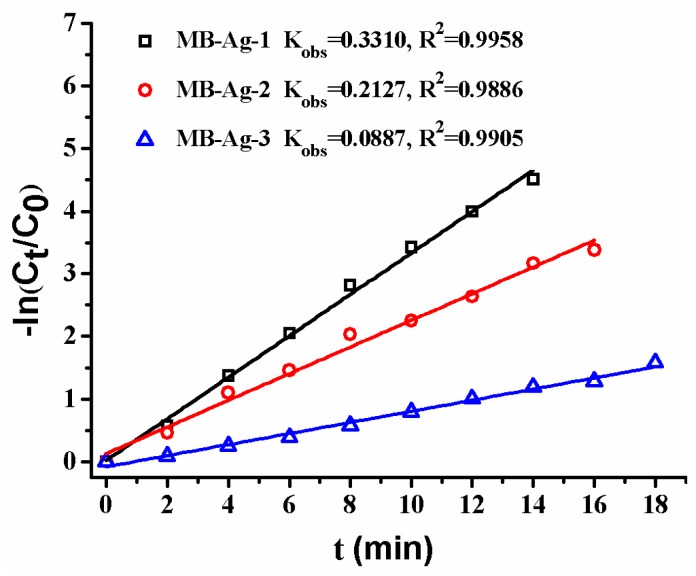
Effect of Ag loading content on catalytic rate.

Previous studies have shown that the contents and specific surface areas of noble metals in catalytic membranes have a great influence on the catalytic reaction [1,2,5,13,17]. In this study, the Ag coating content is also focused on. Figure 12 shows the reduction of *p*-nitrophenol with different composite membranes. A good linear relation of −ln(*C_t_*/*C*_0_) versus reaction time is observed in all reactions, revealing that the reduction of *p*-nitrophenol catalyzed by Ag NPs follows the first-order kinetics [2,13,17]. Furthermore, the reduction rate markedly increases with an increase in Ag content, which is consistent with other noble metal catalysts [2,17]. This is expected, because a greater Ag NP loading on the membrane results in an increase of the surface area of the catalysts, and thus provides more accessible active sites for reactants.

#### 3.4.5. Temperature Dependence and Activation Energy Calculation

**Figure 13:**
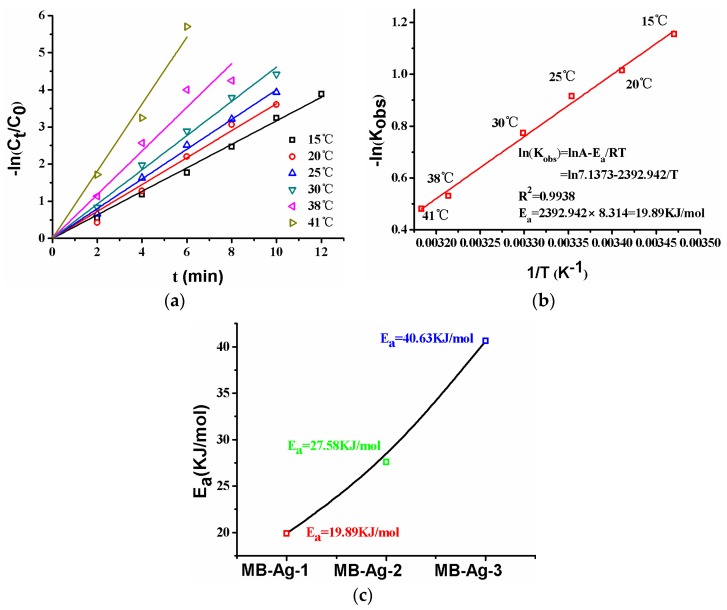
Reactions at different temperatures ((**a**), MB-Ag-1 membrane), *E_a_* ((**b**), MB-Ag-1 membrane), *E*_a_ ((**c**), various membranes). RT: room temperature.

The effect of temperature on the catalytic reduction of *p*-nitrophenol is shown in Figure 13. As can be seen from Figure 13a, the plot of −ln(*C_t_*/*C*_0_) versus reaction time at different temperatures presents linear relationships, and the reduction rate increases with increasing temperature. The specific relationship between the reaction rate constant and temperature can be described by the Arrhenius equation:

(5) $$\ln(k_{obs})=\ln A_{0}-\frac{E_{a}}{RT},$$

where *T* (K) is the thermodynamic temperature, *E_a_* (kJ/mol) is the apparent activation energy, *A*_0_ (min^−1^) is the pre-exponential factor, and *R* is the ideal gas constant. According to the Arrhenius equation, the activation energy of the reaction could be calculated. Normally, the diffusion-controlled mechanism and the surface-controlled mechanism in a reaction are expressed with a lower activation energy (*E_a_*: 8.0–21.0 kJ/mol) and a higher activation energy (*E_a_* > 29.0 kJ/mol), respectively [2]. The activation energy values for different composite membranes are shown in Figure 13c. It is obvious that the activation energy value (*E_a_* = 40.63 kJ/mol) is largest for the composite membrane with the lowest Ag content (MB-Ag-3); with an increase in Ag content, the *E_a_* gradually decreases: the value of *E_a_* is 27.58 kJ/mol (MB-Ag-2) and 19.89 kJ/mol (MB-Ag-1), respectively. Therefore, the reaction shows the diffusion-controlled mechanism for the composite membrane with a high Ag content and the surface-controlled mechanism for the composite membrane with a low Ag content.

### 3.5. Reusability of the Composite Membrane

**Figure 14:**
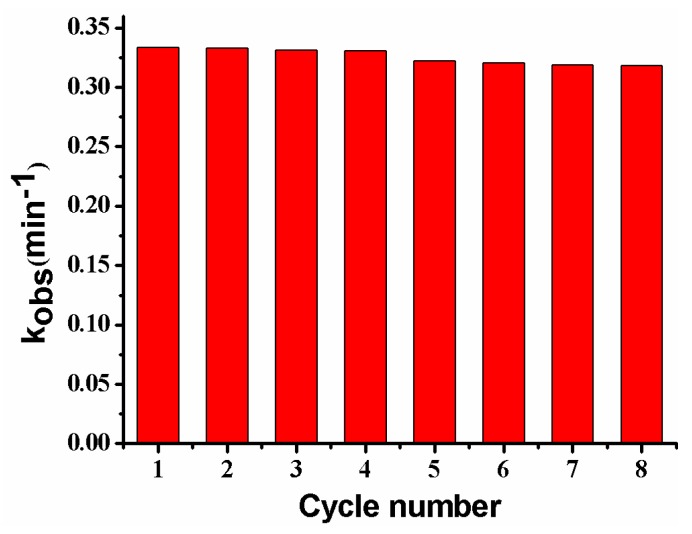
Reusability of the membrane: MB-Ag-1.

Figure 14 shows the reusability of the composite membrane for the catalytic reduction of *p*-nitrophenol. The *k_obs_* almost remains unchanged after reuse for four cycles, and only shows a slight decrease from the 5th to the 8th cycle, possibly caused by the passivation or the loss of a very few Ag NPs in the repeated washing process [2,13]. The result suggests that the PVDF/SiO_2_@Ag membrane exhibits good stability and an antifouling property. Furthermore, since the general steps for the dispersion and separation of metal catalysts are omitted, the operations for using and recovering catalysts are very convenient [2,11].

### 3.6. Separation of Products from Reactants

**Figure 15:**
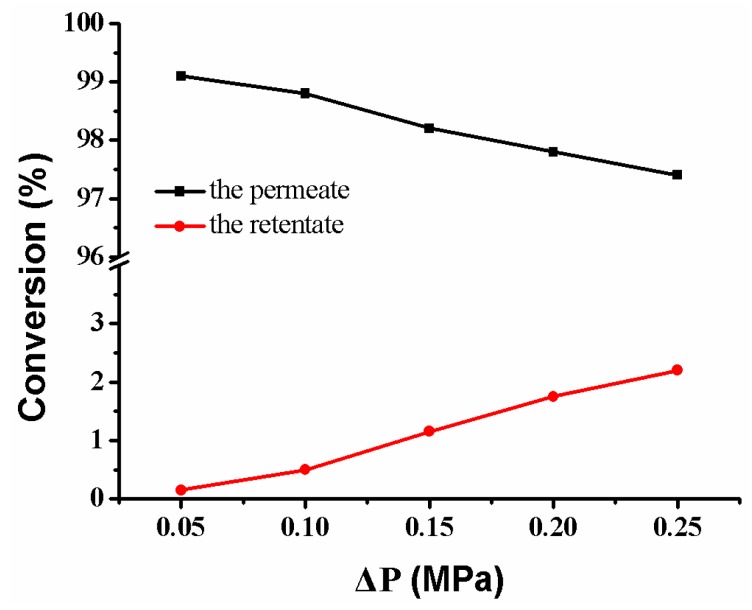
Conversion versus operating pressure in the reduction of *p*-nitrophenol membrane: MB-Ag-1.

Under the cross-flow model, the feed solution passing through the composite membrane includes the retentate on the membrane’s surface and the permeate in the membrane’s pores. Therefore, the reactant’s conversion comes from the retentate and the permeate, respectively. As the pressure increases, the conversion of reactant from the permeate decreases slightly, while the conversion rate of reactant from the retentate increases (Figure 15). The conversion of reactant is closely related to flow rate (Figure 11b). The fast flow rate of the reactant in the retentate and the few Ag NPs on the membrane’s surface make the reactant to be rarely reduced (lower than 3.0%). On the contrary, the slow flow rate of the reactant in the permeate and the abundant Ag NPs in the membrane pores make the reactant be reduced completely (more than 97.5%). Thus, it is possible to directly obtain a high-purity product by collecting the fluid of the permeate [13,17]. Therefore, the separation of the product from the reactant is achieved without the need for additional operating costs and energy consumption.

## 4. Conclusions

This study reported a novel composite membrane with a SiO_2_ microsphere-free porous surface and inner pores with immobilized SiO_2_ microspheres decorated by Ag NPs. The composite membrane was used for the catalytic reduction of *p*-nitrophenol under a cross-flow operating model. It was surprising to find that very high conversion was obtained from the permeate while only very low conversion was obtained from the retentate. The high conversion corresponded to the enriched Ag NPs in the membrane pore, which provides an abundance of reactive sites for reactants, while the low conversion was related to the lack of catalysts on the membrane’s surface. Thus, the products can be separated from the reactants flowing on the membrane’s surface and directly obtained by collecting the fluid of the permeate by using the as-prepared composite membrane. The reaction obeys the first-order kinetics and the rate increases with an increase in temperature, Ag NPs content, and operating pressure. In addition, the composite membrane can be conveniently used and is very stable and therefore can hopefully be used in a wide range of applications.

## Figures and Tables

**Table 1:** Structures and compositions of various composite membranes.

Sample	Average pore size (nm)	Porosity (%)	Ag loading (μg/cm^2^)	Contact angle (°)
PVDF/SiO_2_	120.0 ± 4	70.1	0	83.5
MB-Ag-1	125.0 ± 6	71.2	59.960	81.5
MB-Ag-2	125.0 ± 3	71.2	16.789	80.8
MB-Ag-3	125.0 ± 5	71.2	5.321	80.2
